# (2-Hy­droxy­acetato-κ*O* ^1^)bis­(1,10-phenan­throline-κ^2^ *N*,*N*′)copper(II) nitrate

**DOI:** 10.1107/S1600536811013110

**Published:** 2011-04-13

**Authors:** Ya-Jie Kong, Zhuang-Dong Yuan

**Affiliations:** a Department of Chemistry and Chemical Engineering, Jining University, Qufu 273155, Shandong, People’s Republic of China

## Abstract

In the title compound, [Cu(C_2_H_3_O_3_)(C_12_H_8_N_2_)_2_]NO_3_, the Cu^II^ atom is coordinated by two phenanthroline (phen) ligands and one carboxyl-O atom of a hy­droxy­acetate anion in a distorted square-pyramidal geometry. The hy­droxy group of the hy­droxy­acetate ligand links with the counter NO_3_^−^ anion *via* a pair of bifurcated O—H⋯O hydrogen bonds. The centroid–centroid distance of 3.5676 (14) Å between benzene rings of parallel phen ligands of adjacent mol­ecules suggests the existence of π–π stacking. Weak inter­molecular C—H⋯O hydrogen bonding is also present in the crystal structure.

## Related literature

For related structures, see: Carballo *et al.* (2001 ▶). 
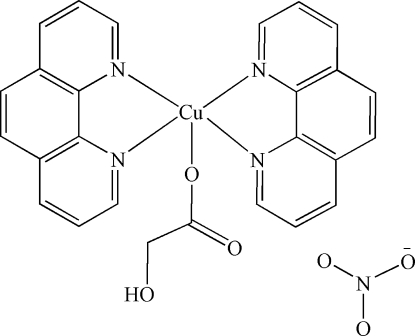


## Experimental

### 

#### Crystal data

[Cu(C_2_H_3_O_3_)(C_12_H_8_N_2_)_2_]NO_3_
*M* *_r_* = 561.00
Monoclinic, 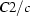

*a* = 21.718 (4) Å
*b* = 14.347 (3) Å
*c* = 16.311 (3) Å
β = 117.045 (3)°
*V* = 4526.5 (16) Å^3^
*Z* = 8
Mo *K*α radiation
μ = 1.02 mm^−1^
*T* = 293 K
0.50 × 0.40 × 0.40 mm

#### Data collection

Bruker SMART 1000 diffractometer
Absorption correction: multi-scan (*SADABS*; Sheldrick, 2004 ▶) *T*_min_ = 0.599, *T*_max_ = 0.664
17009 measured reflections
4425 independent reflections
4047 reflections with *I* > 2σ(*I*)
*R*_int_ = 0.024

#### Refinement

*R*[*F* ^2^ > 2σ(*F* ^2^)] = 0.033
*wR*(*F* ^2^) = 0.101
*S* = 1.06
4425 reflections
343 parameters
H-atom parameters constrained
Δρ_max_ = 0.53 e Å^−3^
Δρ_min_ = −0.23 e Å^−3^

### 

Data collection: *SMART* (Bruker, 2001 ▶); cell refinement: *SAINT* (Bruker, 2001 ▶); data reduction: *SAINT*; program(s) used to solve structure: *SHELXTL* (Sheldrick, 2008 ▶); program(s) used to refine structure: *SHELXTL*; molecular graphics: *SHELXTL*; software used to prepare material for publication: *SHELXTL*.

## Supplementary Material

Crystal structure: contains datablocks global, I. DOI: 10.1107/S1600536811013110/xu5175sup1.cif

Structure factors: contains datablocks I. DOI: 10.1107/S1600536811013110/xu5175Isup2.hkl

Additional supplementary materials:  crystallographic information; 3D view; checkCIF report

## Figures and Tables

**Table 1:** Selected bond lengths (Å)

Cu1—O4	1.9511 (15)
Cu1—N1	2.0109 (16)
Cu1—N2	2.2298 (16)
Cu1—N3	2.0037 (16)
Cu1—N4	2.0449 (15)

**Table 2:** Hydrogen-bond geometry (Å, °)

*D*—H⋯*A*	*D*—H	H⋯*A*	*D*⋯*A*	*D*—H⋯*A*
O6—H6*A*⋯O1	0.82	2.15	2.963 (3)	168
O6—H6*A*⋯O3	0.82	2.49	3.135 (4)	137
C7—H7*A*⋯O3^i^	0.93	2.42	3.351 (3)	176
C16—H16*A*⋯O1^ii^	0.93	2.53	3.383 (4)	152
C18—H18*A*⋯O3^iii^	0.93	2.53	3.456 (4)	172
C26—H26*A*⋯O6^iv^	0.97	2.44	3.297 (3)	147

Symmetry codes: (i) 
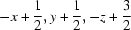
; (ii) 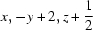; (iii) 
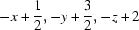
; (iv) 
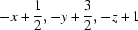
.

**Fig. 1.**
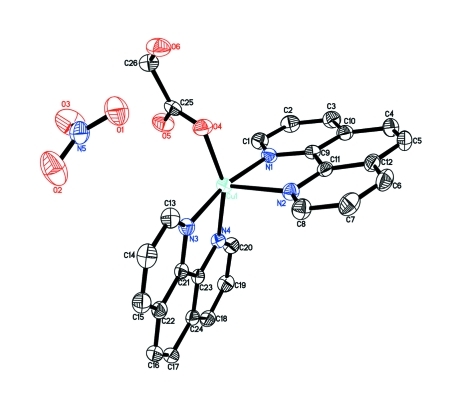
Molecular structure of the title compound drawn with displacement ellipsoids at the 30% probability level. All hydrogen atoms have been omitted for clarity.

**Fig. 2.**
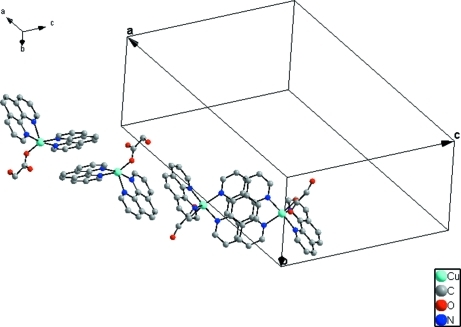
Part of an one-dimensional linear chains of the title compound.

## supplementary crystallographic information

### Comment

The molecules [Cu(C_12_H_8_N_2_)_2_(C_2_H_3_O_3_)] in three different solvents or anions(2-hydroxyacetate anion, 2-hydroxyacetic acid and acetonitrile solvent) have been reported (Carballo *et al.*, 2001).

Crystals [Cu(C_12_H_8_N_2_)_2_(C_2_H_3_O_3_)]NO_3_ (I) were obtained by crystallized from ethanol-water solution. The molecular structure of (I) is shown in Fig. 1. In the title compound the Cu^II^ atom is coordinated by two phenanthroline (phen) ligands and one carboxyl-O atom of a hydroxyacetate anion in a distorted square-pyramidal geometry (Table 1). The hydroxy group of the hydroxyacetate ligand links with the counter NO_3_^-^ anion via a pair of bifurcated O—H···O hydrogen bonds (Table 2). The centroid-to-centroid distance of 3.5676 (14) Å between benzene rings of parallel phen ligands of adjacent molecules suggests the existence of π-π stacking. Weak intermolecular C—H···O hydrogen bonding is also present in the crystal structure.

### Experimental

Copper nitrate (0.093 g, 0.5 mmol) was added to a mixed solution of hydroxyacetic acid (0.076 g, 1 mmol) in distilled water (10 ml) and 1,10-phenanthroline (0.090 g, 0.5 mmol) in ethanol (5 ml). The pH value of the mixture was adjusted to 7 with ammonia. The resulting solution was stirred for 1 h, and then filtered off. The filtrate was left to evaporate showly at room temperature. After a long time, blue block crystals were obtained.

### Refinement

H atoms were positioned geometrically with C—H = 0.93 and 0.97 Å for aromatic and methylene H atoms, respectively, and constrained to ride on their parent atoms with U_iso_(H) = 1.2U_eq_(C).

### Crystal data

**Table:**

[Cu(C_2_H_3_O_3_)(C_12_H_8_N_2_)_2_]NO_3_	*F*(000) = 2296
*M**_r_* = 561.00	*D*_x_ = 1.646 Mg m^−^^3^
Monoclinic, *C*2/*c*	Mo *K*α radiation, λ = 0.71073 Å
Hall symbol: -C 2yc	Cell parameters from 5502 reflections
*a* = 21.718 (4) Å	θ = 2.5–28.2°
*b* = 14.347 (3) Å	µ = 1.02 mm^−^^1^
*c* = 16.311 (3) Å	*T* = 293 K
β = 117.045 (3)°	Block, blue
*V* = 4526.5 (16) Å^3^	0.50 × 0.40 × 0.40 mm
*Z* = 8	

### Data collection

**Table:**

Bruker SMART 1000 diffractometer	4425 independent reflections
Radiation source: fine-focus sealed tube	4047 reflections with *I* > 2σ(*I*)
graphite	*R*_int_ = 0.024
ω–scan	θ_max_ = 26.0°, θ_min_ = 1.8°
Absorption correction: multi-scan (*SADABS*; Sheldrick, 2004)	*h* = −26→26
*T*_min_ = 0.599, *T*_max_ = 0.664	*k* = −17→17
17009 measured reflections	*l* = −20→20

### Refinement

**Table:**

Refinement on *F*^2^	Primary atom site location: structure-invariant direct methods
Least-squares matrix: full	Secondary atom site location: difference Fourier map
*R*[*F*^2^ > 2σ(*F*^2^)] = 0.033	Hydrogen site location: inferred from neighbouring sites
*wR*(*F*^2^) = 0.101	H-atom parameters constrained
*S* = 1.06	*w* = 1/[σ^2^(*F*_o_^2^) + (0.0711*P*)^2^ + 1.7595*P*] where *P* = (*F*_o_^2^ + 2*F*_c_^2^)/3
4425 reflections	(Δ/σ)_max_ = 0.002
343 parameters	Δρ_max_ = 0.53 e Å^−^^3^
0 restraints	Δρ_min_ = −0.23 e Å^−^^3^

### Special details

**Table:**

Geometry. All e.s.d.'s (except the e.s.d. in the dihedral angle between two l.s. planes) are estimated using the full covariance matrix. The cell e.s.d.'s are taken into account individually in the estimation of e.s.d.'s in distances, angles and torsion angles; correlations between e.s.d.'s in cell parameters are only used when they are defined by crystal symmetry. An approximate (isotropic) treatment of cell e.s.d.'s is used for estimating e.s.d.'s involving l.s. planes.
Refinement. Refinement of *F*^2^ against ALL reflections. The weighted *R*-factor *wR* and goodness of fit *S* are based on *F*^2^, conventional *R*-factors *R* are based on *F*, with *F* set to zero for negative *F*^2^. The threshold expression of *F*^2^ > σ(*F*^2^) is used only for calculating *R*-factors(gt) *etc*. and is not relevant to the choice of reflections for refinement. *R*-factors based on *F*^2^ are statistically about twice as large as those based on *F*, and *R*- factors based on ALL data will be even larger.

### Fractional atomic coordinates and isotropic or equivalent isotropic displacement parameters (Å^2^)

**Table:**

	*x*	*y*	*z*	*U*_iso_*/*U*_eq_	
Cu1	0.352559 (11)	0.810948 (15)	0.864386 (15)	0.03488 (11)	
C2	0.51483 (12)	0.63868 (16)	0.86753 (16)	0.0520 (5)	
H2A	0.5233	0.5752	0.8673	0.062*	
C12	0.51725 (10)	0.98715 (15)	0.87161 (13)	0.0431 (4)	
C23	0.33565 (9)	0.80523 (11)	1.02687 (13)	0.0328 (4)	
C11	0.47143 (9)	0.91941 (13)	0.87363 (12)	0.0352 (4)	
C24	0.34342 (10)	0.78875 (14)	1.11575 (13)	0.0374 (4)	
C13	0.23293 (10)	0.93864 (14)	0.82592 (14)	0.0442 (4)	
H13A	0.2283	0.9456	0.7667	0.053*	
C25	0.27347 (10)	0.74094 (14)	0.69714 (13)	0.0403 (4)	
C9	0.48683 (10)	0.82262 (13)	0.87109 (12)	0.0356 (4)	
C21	0.28533 (9)	0.86956 (12)	0.96824 (12)	0.0338 (4)	
C17	0.30025 (11)	0.83895 (17)	1.14532 (14)	0.0458 (5)	
H17A	0.3049	0.8286	1.2041	0.055*	
C14	0.19071 (11)	0.99126 (15)	0.85214 (16)	0.0508 (5)	
H14A	0.1590	1.0331	0.8110	0.061*	
C22	0.24435 (9)	0.91847 (13)	0.99943 (14)	0.0396 (4)	
C20	0.42130 (10)	0.70152 (13)	1.04555 (14)	0.0406 (4)	
H20A	0.4485	0.6717	1.0229	0.049*	
C19	0.43112 (11)	0.67978 (14)	1.13422 (15)	0.0454 (5)	
H19A	0.4638	0.6354	1.1690	0.055*	
C1	0.45271 (12)	0.66987 (14)	0.86349 (15)	0.0444 (5)	
H1A	0.4197	0.6262	0.8589	0.053*	
C8	0.39708 (12)	1.02971 (15)	0.87769 (16)	0.0486 (5)	
H8A	0.3566	1.0450	0.8807	0.058*	
C18	0.39312 (11)	0.72315 (15)	1.16947 (13)	0.0442 (4)	
H18A	0.4000	0.7095	1.2288	0.053*	
C10	0.54928 (11)	0.79679 (15)	0.87236 (14)	0.0428 (4)	
C6	0.49801 (12)	1.07989 (15)	0.87063 (15)	0.0524 (5)	
H6B	0.5262	1.1272	0.8676	0.063*	
C16	0.25344 (10)	0.90029 (15)	1.09058 (14)	0.0464 (5)	
H16A	0.2262	0.9318	1.1120	0.056*	
C15	0.19623 (11)	0.98105 (14)	0.93763 (16)	0.0478 (5)	
H15A	0.1680	1.0156	0.9553	0.057*	
C26	0.22641 (12)	0.73763 (16)	0.59474 (14)	0.0500 (5)	
H26A	0.2470	0.6967	0.5666	0.060*	
H26B	0.1828	0.7101	0.5848	0.060*	
C4	0.59588 (11)	0.86792 (17)	0.87278 (15)	0.0525 (5)	
H4A	0.6378	0.8512	0.8744	0.063*	
C5	0.57971 (11)	0.95780 (17)	0.87093 (16)	0.0541 (5)	
H5A	0.6100	1.0026	0.8691	0.065*	
C7	0.43853 (12)	1.10155 (15)	0.87403 (16)	0.0548 (5)	
H7A	0.4257	1.1634	0.8739	0.066*	
C3	0.56297 (12)	0.70205 (16)	0.87188 (17)	0.0512 (5)	
H3A	0.6046	0.6822	0.8745	0.061*	
O4	0.30007 (8)	0.81909 (10)	0.73072 (10)	0.0469 (4)	
O5	0.28331 (10)	0.66929 (11)	0.74203 (12)	0.0583 (4)	
O6	0.21298 (10)	0.82300 (12)	0.54982 (11)	0.0608 (4)	
H6A	0.1892	0.8549	0.5662	0.091*	
O1	0.12811 (12)	0.95952 (15)	0.59033 (16)	0.0832 (6)	
O3	0.10001 (14)	0.82669 (13)	0.61923 (19)	0.0869 (7)	
O2	0.05765 (12)	0.94510 (15)	0.6477 (2)	0.0971 (8)	
N4	0.37498 (8)	0.76262 (10)	0.99287 (10)	0.0341 (3)	
N1	0.43912 (8)	0.75916 (11)	0.86593 (10)	0.0372 (3)	
N2	0.41221 (8)	0.94125 (11)	0.87707 (11)	0.0391 (3)	
N3	0.27921 (8)	0.87939 (11)	0.88243 (10)	0.0366 (3)	
N5	0.09405 (10)	0.91099 (13)	0.61673 (14)	0.0516 (4)	

### Atomic displacement parameters (Å^2^)

**Table:**

	*U*^11^	*U*^22^	*U*^33^	*U*^12^	*U*^13^	*U*^23^
Cu1	0.03761 (16)	0.03582 (16)	0.03405 (16)	0.00062 (8)	0.01877 (12)	0.00072 (8)
C2	0.0573 (12)	0.0443 (11)	0.0552 (13)	0.0108 (10)	0.0263 (10)	−0.0026 (9)
C12	0.0409 (10)	0.0465 (11)	0.0359 (10)	−0.0102 (8)	0.0122 (8)	−0.0016 (8)
C23	0.0316 (8)	0.0329 (9)	0.0347 (9)	−0.0083 (6)	0.0159 (7)	−0.0041 (6)
C11	0.0379 (9)	0.0373 (9)	0.0278 (8)	−0.0050 (7)	0.0127 (7)	−0.0013 (7)
C24	0.0376 (9)	0.0391 (9)	0.0365 (9)	−0.0110 (8)	0.0178 (8)	−0.0041 (7)
C13	0.0436 (10)	0.0413 (10)	0.0450 (10)	0.0025 (8)	0.0178 (9)	0.0084 (8)
C25	0.0436 (10)	0.0472 (11)	0.0406 (10)	0.0035 (8)	0.0284 (8)	0.0006 (8)
C9	0.0373 (9)	0.0403 (10)	0.0286 (9)	−0.0014 (7)	0.0145 (7)	−0.0008 (7)
C21	0.0317 (8)	0.0327 (9)	0.0373 (9)	−0.0066 (7)	0.0158 (7)	−0.0043 (7)
C17	0.0458 (11)	0.0577 (12)	0.0412 (11)	−0.0122 (10)	0.0263 (9)	−0.0100 (9)
C14	0.0448 (11)	0.0360 (10)	0.0650 (14)	0.0057 (8)	0.0191 (10)	0.0062 (9)
C22	0.0349 (9)	0.0360 (9)	0.0489 (10)	−0.0049 (7)	0.0199 (8)	−0.0097 (8)
C20	0.0391 (10)	0.0395 (10)	0.0410 (10)	0.0030 (8)	0.0165 (8)	0.0013 (8)
C19	0.0464 (11)	0.0420 (11)	0.0402 (11)	0.0017 (8)	0.0129 (9)	0.0069 (8)
C1	0.0518 (12)	0.0350 (10)	0.0487 (12)	0.0000 (8)	0.0247 (10)	−0.0013 (8)
C8	0.0491 (11)	0.0383 (11)	0.0569 (12)	0.0001 (9)	0.0228 (10)	−0.0034 (9)
C18	0.0478 (11)	0.0484 (11)	0.0338 (9)	−0.0102 (9)	0.0162 (8)	0.0029 (8)
C10	0.0390 (10)	0.0529 (11)	0.0356 (10)	0.0004 (8)	0.0162 (8)	−0.0034 (8)
C6	0.0542 (12)	0.0420 (11)	0.0502 (12)	−0.0171 (9)	0.0145 (10)	−0.0017 (9)
C16	0.0432 (10)	0.0530 (12)	0.0498 (11)	−0.0073 (9)	0.0271 (9)	−0.0155 (9)
C15	0.0431 (10)	0.0380 (10)	0.0617 (13)	0.0004 (8)	0.0234 (10)	−0.0086 (9)
C26	0.0539 (12)	0.0556 (13)	0.0425 (11)	−0.0028 (10)	0.0237 (9)	−0.0058 (9)
C4	0.0373 (10)	0.0688 (15)	0.0522 (12)	−0.0069 (10)	0.0210 (9)	−0.0061 (10)
C5	0.0443 (11)	0.0633 (14)	0.0541 (13)	−0.0191 (10)	0.0218 (10)	−0.0041 (10)
C7	0.0629 (13)	0.0337 (10)	0.0582 (13)	−0.0044 (9)	0.0190 (11)	−0.0027 (9)
C3	0.0441 (11)	0.0582 (13)	0.0515 (13)	0.0086 (9)	0.0218 (10)	−0.0042 (10)
O4	0.0547 (9)	0.0491 (9)	0.0351 (7)	−0.0018 (6)	0.0188 (7)	−0.0006 (6)
O5	0.0761 (11)	0.0511 (9)	0.0560 (10)	0.0039 (8)	0.0374 (9)	0.0108 (7)
O6	0.0698 (11)	0.0724 (11)	0.0412 (8)	0.0117 (8)	0.0261 (8)	0.0105 (7)
O1	0.0908 (14)	0.0763 (13)	0.0973 (15)	−0.0311 (11)	0.0556 (13)	0.0017 (11)
O3	0.127 (2)	0.0480 (11)	0.1166 (19)	0.0048 (11)	0.0819 (17)	0.0051 (10)
O2	0.0933 (15)	0.0659 (13)	0.164 (3)	0.0014 (11)	0.0865 (18)	−0.0056 (13)
N4	0.0343 (7)	0.0347 (8)	0.0336 (7)	−0.0011 (6)	0.0156 (6)	−0.0004 (6)
N1	0.0416 (8)	0.0360 (8)	0.0384 (8)	−0.0003 (6)	0.0222 (7)	−0.0027 (6)
N2	0.0425 (8)	0.0340 (8)	0.0408 (8)	−0.0026 (6)	0.0189 (7)	−0.0018 (6)
N3	0.0356 (7)	0.0359 (8)	0.0383 (8)	−0.0005 (6)	0.0168 (6)	0.0021 (6)
N5	0.0493 (10)	0.0467 (10)	0.0585 (11)	−0.0076 (8)	0.0243 (9)	−0.0015 (8)

### Geometric parameters (Å, °)

**Table:**

Cu1—O4	1.9511 (15)	C22—C15	1.399 (3)
Cu1—N1	2.0109 (16)	C22—C16	1.432 (3)
Cu1—N2	2.2298 (16)	C20—N4	1.316 (2)
Cu1—N3	2.0037 (16)	C20—C19	1.398 (3)
Cu1—N4	2.0449 (15)	C20—H20A	0.9300
C2—C3	1.363 (4)	C19—C18	1.353 (3)
C2—C1	1.394 (3)	C19—H19A	0.9300
C2—H2A	0.9300	C1—N1	1.319 (3)
C12—C6	1.393 (3)	C1—H1A	0.9300
C12—C11	1.402 (3)	C8—N2	1.312 (3)
C12—C5	1.425 (3)	C8—C7	1.387 (3)
C23—N4	1.357 (2)	C8—H8A	0.9300
C23—C24	1.401 (3)	C18—H18A	0.9300
C23—C21	1.418 (3)	C10—C3	1.392 (3)
C11—N2	1.350 (2)	C10—C4	1.435 (3)
C11—C9	1.433 (3)	C6—C7	1.354 (3)
C24—C18	1.400 (3)	C6—H6B	0.9300
C24—C17	1.429 (3)	C16—H16A	0.9300
C13—N3	1.319 (3)	C15—H15A	0.9300
C13—C14	1.397 (3)	C26—O6	1.388 (3)
C13—H13A	0.9300	C26—H26A	0.9700
C25—O5	1.223 (3)	C26—H26B	0.9700
C25—O4	1.266 (2)	C4—C5	1.333 (3)
C25—C26	1.511 (3)	C4—H4A	0.9300
C9—N1	1.353 (2)	C5—H5A	0.9300
C9—C10	1.397 (3)	C7—H7A	0.9300
C21—N3	1.352 (2)	C3—H3A	0.9300
C21—C22	1.398 (2)	O6—H6A	0.8200
C17—C16	1.334 (3)	O1—N5	1.228 (3)
C17—H17A	0.9300	O3—N5	1.215 (3)
C14—C15	1.352 (3)	O2—N5	1.217 (3)
C14—H14A	0.9300		
			
O4—Cu1—N3	92.02 (6)	C2—C1—H1A	118.8
O4—Cu1—N1	95.97 (6)	N2—C8—C7	123.3 (2)
N3—Cu1—N1	168.63 (6)	N2—C8—H8A	118.4
O4—Cu1—N4	155.70 (6)	C7—C8—H8A	118.4
N3—Cu1—N4	81.39 (6)	C19—C18—C24	119.28 (18)
N1—Cu1—N4	94.38 (6)	C19—C18—H18A	120.4
O4—Cu1—N2	94.19 (6)	C24—C18—H18A	120.4
N3—Cu1—N2	92.45 (6)	C3—C10—C9	117.9 (2)
N1—Cu1—N2	78.94 (6)	C3—C10—C4	122.8 (2)
N4—Cu1—N2	109.36 (6)	C9—C10—C4	119.3 (2)
C3—C2—C1	119.4 (2)	C7—C6—C12	120.4 (2)
C3—C2—H2A	120.3	C7—C6—H6B	119.8
C1—C2—H2A	120.3	C12—C6—H6B	119.8
C6—C12—C11	116.7 (2)	C17—C16—C22	121.22 (18)
C6—C12—C5	124.3 (2)	C17—C16—H16A	119.4
C11—C12—C5	118.9 (2)	C22—C16—H16A	119.4
N4—C23—C24	123.01 (17)	C14—C15—C22	119.98 (19)
N4—C23—C21	116.93 (16)	C14—C15—H15A	120.0
C24—C23—C21	120.06 (17)	C22—C15—H15A	120.0
N2—C11—C12	122.69 (18)	O6—C26—C25	115.45 (19)
N2—C11—C9	117.74 (16)	O6—C26—H26A	108.4
C12—C11—C9	119.57 (18)	C25—C26—H26A	108.4
C18—C24—C23	117.19 (18)	O6—C26—H26B	108.4
C18—C24—C17	124.43 (18)	C25—C26—H26B	108.4
C23—C24—C17	118.38 (19)	H26A—C26—H26B	107.5
N3—C13—C14	122.1 (2)	C5—C4—C10	120.6 (2)
N3—C13—H13A	118.9	C5—C4—H4A	119.7
C14—C13—H13A	118.9	C10—C4—H4A	119.7
O5—C25—O4	124.3 (2)	C4—C5—C12	121.8 (2)
O5—C25—C26	118.8 (2)	C4—C5—H5A	119.1
O4—C25—C26	116.88 (18)	C12—C5—H5A	119.1
N1—C9—C10	122.23 (18)	C6—C7—C8	118.7 (2)
N1—C9—C11	118.14 (17)	C6—C7—H7A	120.6
C10—C9—C11	119.62 (17)	C8—C7—H7A	120.6
N3—C21—C22	123.04 (17)	C2—C3—C10	119.4 (2)
N3—C21—C23	116.68 (16)	C2—C3—H3A	120.3
C22—C21—C23	120.28 (17)	C10—C3—H3A	120.3
C16—C17—C24	121.60 (19)	C25—O4—Cu1	110.50 (12)
C16—C17—H17A	119.2	C26—O6—H6A	109.5
C24—C17—H17A	119.2	C20—N4—C23	118.00 (16)
C15—C14—C13	119.6 (2)	C20—N4—Cu1	130.53 (13)
C15—C14—H14A	120.2	C23—N4—Cu1	111.43 (12)
C13—C14—H14A	120.2	C1—N1—C9	118.67 (17)
C21—C22—C15	116.77 (19)	C1—N1—Cu1	125.39 (14)
C21—C22—C16	118.44 (18)	C9—N1—Cu1	115.93 (12)
C15—C22—C16	124.77 (18)	C8—N2—C11	118.14 (17)
N4—C20—C19	122.40 (19)	C8—N2—Cu1	132.42 (15)
N4—C20—H20A	118.8	C11—N2—Cu1	109.03 (12)
C19—C20—H20A	118.8	C13—N3—C21	118.41 (17)
C18—C19—C20	120.10 (19)	C13—N3—Cu1	128.11 (14)
C18—C19—H19A	120.0	C21—N3—Cu1	112.99 (12)
C20—C19—H19A	120.0	O3—N5—O2	117.7 (2)
N1—C1—C2	122.4 (2)	O3—N5—O1	120.3 (2)
N1—C1—H1A	118.8	O2—N5—O1	121.7 (2)

### Hydrogen-bond geometry (Å, °)

**Table:**

*D*—H···*A*	*D*—H	H···*A*	*D*···*A*	*D*—H···*A*
O6—H6A···O1	0.82	2.15	2.963 (3)	168
O6—H6A···O3	0.82	2.49	3.135 (4)	137
C7—H7A···O3^i^	0.93	2.42	3.351 (3)	176
C16—H16A···O1^ii^	0.93	2.53	3.383 (4)	152
C18—H18A···O3^iii^	0.93	2.53	3.456 (4)	172
C26—H26A···O6^iv^	0.97	2.44	3.297 (3)	147

Symmetry codes: (i) −*x*+1/2, *y*+1/2, −*z*+3/2; (ii) *x*, −*y*+2, *z*+1/2; (iii) −*x*+1/2, −*y*+3/2, −*z*+2; (iv) −*x*+1/2, −*y*+3/2, −*z*+1.
